# Buyang Huanwu Decoction alleviates cerebral ischemic injury through modulating caveolin-1-mediated mitochondrial quality control

**DOI:** 10.3389/fphar.2023.1137609

**Published:** 2023-05-10

**Authors:** Yaqian Xu, Bowei Chen, Jian Yi, Fengming Tian, Yingfei Liu, Yin Ouyang, Chunyun Yuan, Baiyan Liu

**Affiliations:** ^1^ The First Hospital of Hunan University of Chinese Medicine, Changsha, China; ^2^ MOE Key Laboratory of Research and Translation on Prevention and Treatment of Major Diseases in Internal Medicine of Traditional Chinese Medicine, Hunan University of Chinese Medicine, Changsha, China; ^3^ Hunan Hospital of Integrated Traditional Chinese and Western Medicine, Changsha, China; ^4^ Affiliated Hospital of Hunan Academy of Chinese Medicine, Changsha, China; ^5^ Hunan Academy of Chinese Medicine, Changsha, China

**Keywords:** Buyang Huanwu Decoction, caveolin-1, cerebral ischemia, mitochondrial quality control, mitochondrial dynamics, mitophagy, mitochondrial biogenesis

## Abstract

**Introduction:** Mitochondrial quality control (MQC) is an important mechanism of neural repair after cerebral ischemia (CI). Recent studies have shown that caveolin-1 (Cav-1) is an important signaling molecule in the process of CI injury, but its mechanism of regulating MQC after CI is still unclear. Buyang Huanwu Decoction (BHD) is a classic traditional Chinese medicine formula that is often used to treat CI. Unfortunately, its mechanism of action is still obscure.

**Methods:** In this study, we tested the hypothesis that BHD can regulate MQC through Cav-1 and exert an anti-cerebral ischemia injury effect. We used Cav-1 knockout mice and their homologous wild-type mice, replicated middle cerebral artery occlusion (MCAO) model and BHD intervention. Neurobehavioral scores and pathological detection were used to evaluate neurological function and neuron damage, transmission electron microscopy and enzymology detection of mitochondrial damage. Finally, western blot and RT-qPCR expression of MQC-related molecules were tested.

**Results:** After CI, mice showed neurologic impairment, neuronal damage, and significant destruction of mitochondrial morphology and function, and MQC was imbalanced. Cav-1 deletion aggravated the damage to neurological function, neurons, mitochondrial morphology and mitochondrial function after CI, aggravated the imbalance of mitochondrial dynamics, and inhibited mitophagy and biosynthesis. BHD can maintain MQC homeostasis after CI through Cav-1 and improve CI injury.

**Discussion:** Cav-1 can affect CI injury by regulating MQC, and this mechanism may be another target of BHD for anti-cerebral ischemia injury.

## 1 Introduction

Cerebral ischemia (CI) is a common major disease that seriously threatens human health and life. It has high morbidity, disability and mortality rates and places heavy economic and social burdens on patients and their families (GBD Neurology Collaborators, 2019). Nerve injury after CI involves a series of complex pathophysiological mechanisms, including excitotoxicity of amino acids, imbalance of mitochondrial quality control (MQC), inflammatory cascade and oxygen free radical release (Bakthavachalam and Shanmugam, 2017). Among them, the imbalance of MQC is an important mechanism leading to neuronal cell death. Under physiological conditions, mitochondria need to synthesize new mitochondria in time, maintain mitochondrial morphology and remove damaged mitochondria through a quality control system to maintain the stability of the intracellular environment. After CI, an imbalance in MQC can lead to damage to the structure and function of mitochondria and ultimately cause irreversible damage leading to neuronal cell death (Carinci et al., 2021). An increasing number of studies have shown that MQC is essential for the survival of neural cells and the improvement of neural function after CI(Anzell et al., 2018). It is important to treat CI by initiating endogenous neuroprotective mechanisms related to MQC to repair damaged mitochondria and improve the energy metabolism of nerve cells.

Caveolae are special depressions on the cell membrane that accumulate various cell signaling molecules. They are abundantly expressed in vascular endothelial cells and neuronal cells (Krupinski et al., 1994). Caveolin 1 (Cav-1) is an important functional structure and core protein in caveolae. It not only participates in the development of the nervous system but also regulates blood‒brain barrier permeability, inflammation, angiogenesis and nerve regeneration, is involved in CI injury and is considered a new target for CI therapy (Huang et al., 2018). Recent studies have shown that overexpression of Cav-1 can reduce brain edema after CI (Choi et al., 2016), improve functional neuroplasticity, and improve neurological function after CI (Wang and Head, 2019). The above studies suggest that Cav-1 may be a potential target for the treatment of CI.

A series of recent studies have shown that Cav-1 is closely associated with mitochondria. For example, some researchers have found by immunoelectron microscopy that Cav-1-positive vesicles can be found around mitochondrial double membrane structures (Li et al., 2001). Cav-1 also directly regulates the assembly of the mitochondrial respiratory chain (MRC). In fibroblasts, depletion of Cav-1 leads to inhibition of MRC complexes (Pavlides et al., 2010). In addition, recent studies have shown that Cav-1 depletion alters mitochondrial morphology in tumor cells, causes disturbed mitochondrial dynamics, and regulates mitophagy, ultimately affecting cancer cell survival (Jiang et al., 2022). Thus, it is becoming increasingly clear that Cav-1 is an important regulator of mitochondrial structure and function, and it is closely related to MQC. Unfortunately, there are no reports on the effect of Cav-1 on neural repair after CI through regulating MQC.

**FIGURE 1:**
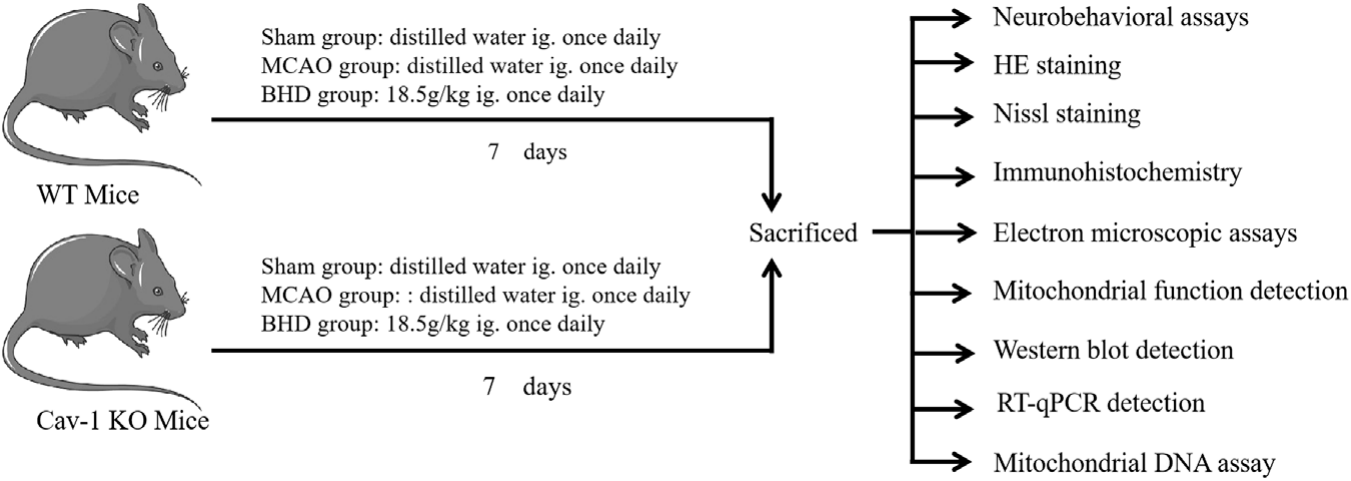
Experimental design.

Buyang Huanwu Decoction (BHD) is a classic prescription of traditional Chinese medicine for the treatment of CI, and its efficacy has been confirmed by evidence-based medicine (Jiang et al., 2020; Shao et al., 2022). Our previous study found that BHD could upregulate the expression of Cav-1 after CI and play an anti-CI role (Liu et al., 2008). However, the mechanism by which BHD promotes nerve repair after CI is not fully understood. In this study, we used Cav-1 knockout (KO) mice and their homologous wild-type (WT) mice to replicate the middle cerebral artery occlusion (MCAO) model and intervened with BHD and finally confirmed that BHD could play a therapeutic role in nerve injury after CI by regulating MQC through Cav-1, as shown in Figure 1.

## 2 Materials and methods

### 2.1 Animals

Specific pathogen-free (SPF) male Cav-1 KO (Cav-1^−/−^) C57BL/6J mice and homologous WT (Cav-1^+/+^) C57BL/6J mice, 6–8 weeks old, weighing 23–28 g. Before the experiment began, Cav-1 heterozygous (Cav-1^+/−^) C57BL/6J mice were obtained from Jiangsu GemPharmatech Co., Ltd. (Strain ID: T010293) and were bred in the SPF animal room of the First Hospital of Hunan University of Chinese Medicine for a long time. The offspring of mice were identified as KO mice and homologous WT mice by PCR and were included in the experiment. The PCR analysis protocol is shown in Supplementary Material S1. This experiment was approved by the experimental animal ethics committee of the First Hospital of Hunan University of Chinese Medicine (ZYFY20201215-1).

### 2.2 Preparation and quality control of BHD

BHD consists of *Astragalus mongholicus* Bunge 120 g, *Angelica sinensis* (Oliv.) Diels 6 g, *Paeonia lactiflora* Pall 4.5 g, *Prunus persica* (L.) Batsch 3 g, *Carthamus tinctorius* L. 3 g, *Pheretima aspergillum* (E. Perrier) 3 g, and *Ligusticum chuanxiong hort* 3 g, which were purchased from the First Hospital of Hunan University of Chinese Medicine. All ingredients were identified as genuine medicinal materials. After all the ingredients were soaked in 5 times distilled water for 1 h, they were decocted with fire for 0.5 h and slow fire for 1.5 h and filtered with 3 layers of gauze. Continuing to add 3 times the amount of distilled water, the filtrate was extracted the same method. After combining the filtrates 2 times, a rotary evaporator was used to prepare the liquid medicine as a concentrated liquid of 2 g/mL based on the amount of crude medicine.

According to our previous report (Chen et al., 2022), we performed ultra-performance liquid chromatography-quadrupole-time of flight-mass spectrometry (UPLC-Q-TOF-MS) analysis on BHD and identified a total of 21 chemical components, of which the mass concentrations of astragaloside IV, formononetin, ferulic acid, and paeoniflorin were 78.1, 46.7, 45.6, and 468.4 μg/mL, respectively.

### 2.3 Reagent

NeuN antibody (Proteintech, 26975-1-AP,Wuhan, China), dynamin 1-like (DRP1) antibody (Boster, A00556-2,Wuhan, China), fission 1 (FIS1) (Boster, A01932-2,Wuhan, China), mitofusin 2 (MFN2) (Boster, BM4906,Wuhan, China), optic atrophy 1 (OPA1) (Boster, PB0773,Wuhan, China), PTEN induced putative kinase 1 (PINK1) (Proteintech, 23274-1-AP,Wuhan, China), PARKIN (Boster, PB9307,Wuhan, China), Beclin 1 (Boster, PB9076,Wuhan, China), microtubule-associated protein 1 light chain 3 beta (LC3) antibody (Proteintech, 14600-1-AP,Wuhan, China), sirtuin 1 (SIRT1) antibody (Boster, A00018-1,Wuhan, China), peroxisome proliferator-activated receptor gamma, coactivator 1 alpha (PGC-1α)Antibody (Proteintech, 66369-1-Ig,Wuhan, China), adenosine triphosphate (ATP) assay kit (Jiancheng, A095-1, Nanjing, China), ATPase assay kit (Jiancheng, A016-1, Nanjing, China), RNA Extraction Kit (Beyotime, R0026, Beijing, China), First Strand cDNA Synthesis Kit (Beijing Beyotime, D7178), Universal Genomic DNA Purification Mini Spin Kit (Beyotime, D0063, Beijing, China), SYBR Green qPCR Mix (Beyotime, product number D7265, Beijing, China), the PCR primers were synthesized by Sangon (Shanghai, China), citrate synthase (CS) assay kit (Solarbio, BC1060, Beijing, China), Nicotinamide adenine dinucleotide (NADH)- coenzyme Q reductase assay kit (Solarbio, BC0510, Beijing, China), succinate-coenzyme Q assay kit (Solarbio, BC3230, Beijing, China), coenzyme Q-cytochrome C assay kit (Solarbio, BC3240, Beijing, China), cytochrome C oxidase assay kit (Solarbio, BC0940, Beijing, China).

### 2.4 Instrument

An intelligent tissue slice imaging system (PerkinElmer, Vectra3, Waltham, MA, United States), transmission electron microscope (Hitachi, HT7700, Tokyo, Japan), nucleic acid protein concentration analyzer (BIO-DROP, K3 TOUCH, Berkeley, CA, United States), fluorescence quantitative PCR instrument (Eppendorf, Realplex2, Hamburg, Germany), chemiluminescence imaging analysis system (Tanon, 5200, Shanghai, China), and multifunctional microplate reader (PerkinElmer, Enspire, Waltham, MA, United States) were used.

### 2.5 Experimental design

KO mice and WT mice were randomly divided into the sham group, model group and BHD group, with 12 mice in each group, to replicate the CI model by MCAO. The mice were anesthetized and fixed. A median neck incision was made. The left common carotid artery, internal carotid artery and external carotid artery were bluntly separated. The suture plug was sent to the internal carotid artery through the common carotid artery. When the black mark point on the suture plug was just located at the bifurcation of the common carotid artery, the suture plug was fixed, and the skin was disinfected and sutured. In the sham group, mice only cut free blood vessels in the skin and then sutured the skin. According to previous reports (Longa et al., 1989), the neurobehavioral score of mice was determined 2 h after the operation, and the model was successfully reproduced if the score was 1–3. According to a previous study, the dosage of BHD was determined to be 18.5 g/kg (Huang et al., 2016; Zhou et al., 2016). The BHD group was given BHD via gastrogavage on the first day after the operation, while the model group and sham group were given an equal volume of distilled water via gastrogavage, gavage volume 0.2 mL/10 g, once a day.

The seventh day after CI is the golden period of brain tissue repair (Ergul et al., 2012; Hatakeyama et al., 2020). Therefore, the neurobehavioral score was determined on the 7th day, and then the mice were anesthetized by intraperitoneal injection of 0.3% sodium pentobarbital and sacrificed. Brain tissues were collected and fixed in 4% paraformaldehyde for HE staining, Nissl staining and immunohistochemical detection. The other part is divided into two parts. One part was quickly placed into liquid nitrogen for freezing and then stored in a −80°C refrigerator for Western blot, PCR and enzymatic detection. One part was cut into 1 mm^3^ tissue blocks, quickly placed into the precooled electron microscope fixative, and stored in a refrigerator at 4°C for transmission electron microscope (TEM) detection.

### 2.6 Neurobehavioral assessment

All mice were subjected to a neurobehavioral assessment on the 7th day after the operation. The neurological function of mice was evaluated by the neurological function score reported by Levy et al. (2009). The higher the score, the more severe the neurological deficit in mice.

### 2.7 HE staining

The whole brain was fixed in 4% paraformaldehyde, dehydrated, transparent, embedded, sectioned and stained with hematoxylin and eosin. After sealing, the whole brain was scanned, and images of the ischemic cortex were selected at ×400 magnification.

### 2.8 Nissl staining

The whole brain was fixed in 4% paraformaldehyde, dehydrated, made transparent, embedded, sectioned, and stained with 1% toluidine blue at 60°C for 40 min. After sectioning, the whole brain was scanned. The images of the ischemic cortex were selected and analyzed by Image-Pro Plus 6.0.

### 2.9 Immunohistochemical staining

Paraffin sections of the whole brain were removed, dewaxed, antigen repaired and blocked in turn, and the rabbit anti-NeuN (1:2000) primary antibody was added and incubated at 4°C overnight. The secondary antibody was added, incubated at 37°C for 1 h, rinsed with PBS and incubated with SABC for 30 min at room temperature. After washing with PBS, the slices were stained with DAB and hematoxylin and finally sealed with glycerol. Whole-section scans were performed, and images of cortical areas on the ischemic side were selected at ×400 magnification. According to our previous report (Tang et al., 2022), images were analyzed with Image-Pro Plus 6.0, and NeuN expression was assessed using the histochemical score (H-score). The H-score was calculated using the following formula: H-score = (percentage of weakly intense cells ×1) + (percentage of moderately intense cells ×2) + (percentage of strongly intense cells ×3).

### 2.10 TEM detection

The cortical tissue on the ischemic side blocks with a volume of approximately 1 mm^3^ were fixed in 2.5% glutaraldehyde solution for 24 h, rinsed with PBS solution three times, fixed in 1% osmic acid fixative solution at 4°C for 2–3 h, dehydrated with gradient ethanol solution, replaced and soaked with acetone, embedded in pure acetone and embedding solution, baked and solidified, and sliced with an ultramicrotome (70 nm). Then, 3% uranyl acetate lead citrate double staining was conducted, and the sections were observed and photographed under a TEM.

The changes in the ultrastructure of mitochondria were evaluated using the Flameng scoring method (Flameng et al., 1980). Three fields of vision were randomly selected for each sample, and the mitochondria in each field were scored according to the degree of ultrastructural changes. Flameng score: 0 point: normal ultrastructural mitochondria, with intact particles; 1 point: the structure is basically normal, and some particles are lost; 2 points: mitochondria were swollen, and the matrix was transparent; 3 points: the matrix was transparent, and the mitochondrial cristae were broken or flocculent aggregation appeared in the matrix; 4 points: loss of matrix, mitochondrial crista rupture, and incomplete outer membrane.

### 2.11 CS and MRC complex activities detection

Approximately 0.1 g of ischemic lateral cortical brain tissue was weighed, and 1.0 mL of extract was added and homogenized on ice. Centrifuge at 3,000 rpm for 10 min. The supernatant was transferred to a separate centrifuge tube and centrifuged at 11,000×*g* for 15 min. Subsequently, 400 μL extracting solution was added into the precipitate, which was subjected to ultrasonic fragmentation. After protein quantification, the CS activity and MRC complex (including NADH-coenzyme Q reductase (complex I), succinate-coenzyme Q (complex II), coenzyme Q-cytochrome C (complex III) and cytochrome C oxidase (complex IV)) activities were measured according to the kit instructions (Liu et al., 2017; Song et al., 2018; Zhu et al., 2018).

### 2.12 ATP content and ATPase activity detection

Appropriate amounts of the cortical tissue on the ischemic side were added to a ratio of weight (g): volume (mL) = 1:9 with precooled distilled water and homogenized in an ice water bath to make a 10% homogenate. The heart was separated at 2,500 rpm for 10 min, and 0.2 mL of the supernatant was diluted into 2% homogenate with 0.8 mL of normal saline. ATP content was determined according to the instructions of the ATP kit, and ATP lysate was diluted to draw a standard curve to calculate ATP content. Na^+^K^+^-ATPase and Ca^2+^Mg^2+^-ATPase activities were measured after enzymatic reaction and phosphorus fixation experiments, and the enzyme concentration was calculated.

### 2.13 Western blot detection

The cortical tissue on the ischemic side was added to RIPA lysis buffer, the supernatant was collected by centrifugation, the total protein was extracted, and the protein concentration was determined by the BCA method. Proteins were denatured by boiling, loaded and subjected to SDS‒PAGE. The membrane was rotated at 300 mA at constant flow for 30 min and blocked with 5% skim milk. Primary antibodies were added (the dilution ratio of the reference protein GAPDH was 1:2000, and the dilution ratio of the target proteins DRP1, FIS1, MFN2, OPA1, PINK1, PARKIN, Beclin 1, LC3, SIRT1 and PGC-1α was 1:1000). HRP-labeled secondary antibody was added at a dilution ratio of 1:5000, incubated at room temperature for 30 min, developed by ECL solution, and imaged by a chemiluminescence system. ImageJ software was used to measure the gray value, and the average gray value was calculated by the gray ratio of the target protein and the internal reference protein bands for relative quantification of protein expression.

### 2.14 RT‒qPCR detection

An appropriate amount of cortical tissue on the ischemic side was placed into a 1.5 mL centrifuge tube, quickly added to precooled lysate, homogenized with a microelectric homogenizer, and RNA was extracted by the centrifugal column method. RNA was reverse transcribed into cDNA. PCR amplification was performed. The primer sequences are detailed in Supplementary Table S1. β-Actin was used as the internal reference gene, and the relative gene expression was calculated using the 2^−ΔΔ CT^ method.

### 2.15 Mitochondrial DNA (mtDNA) assay

An appropriate amount of cortical tissue on the ischemic side was placed into a 1.5 mL centrifuge tube, DNA was extracted by the centrifugal column method, and PCR amplification was performed. Because the gene fragment of NADH dehydrogenase subunit 1 (ND1) is a highly conserved sequence of mtDNA, the ND1 level was selected as the expression amount of mtDNA copy number (Wu et al., 2018). The primer sequences are detailed in Supplementary Table S2. GAPDH was used as the internal reference gene, and the relative gene expression was calculated using the 2^−ΔΔ CT^ method.

### 2.16 Statistical analysis

GraphPad Prism 8.0.1 statistical software was used for analysis and processing. The test data were expressed as the mean ± standard error ( $\bar{x}$ ±s). Multiple-way ANOVA was used for pairwise comparisons between multiple groups, and *p* < 0.05 was considered statistically significant.

## 3 Results

### 3.1 BHD improved neurobehavioral scores and neuronal injury in MCAO mice through Cav-1

**FIGURE 2:**
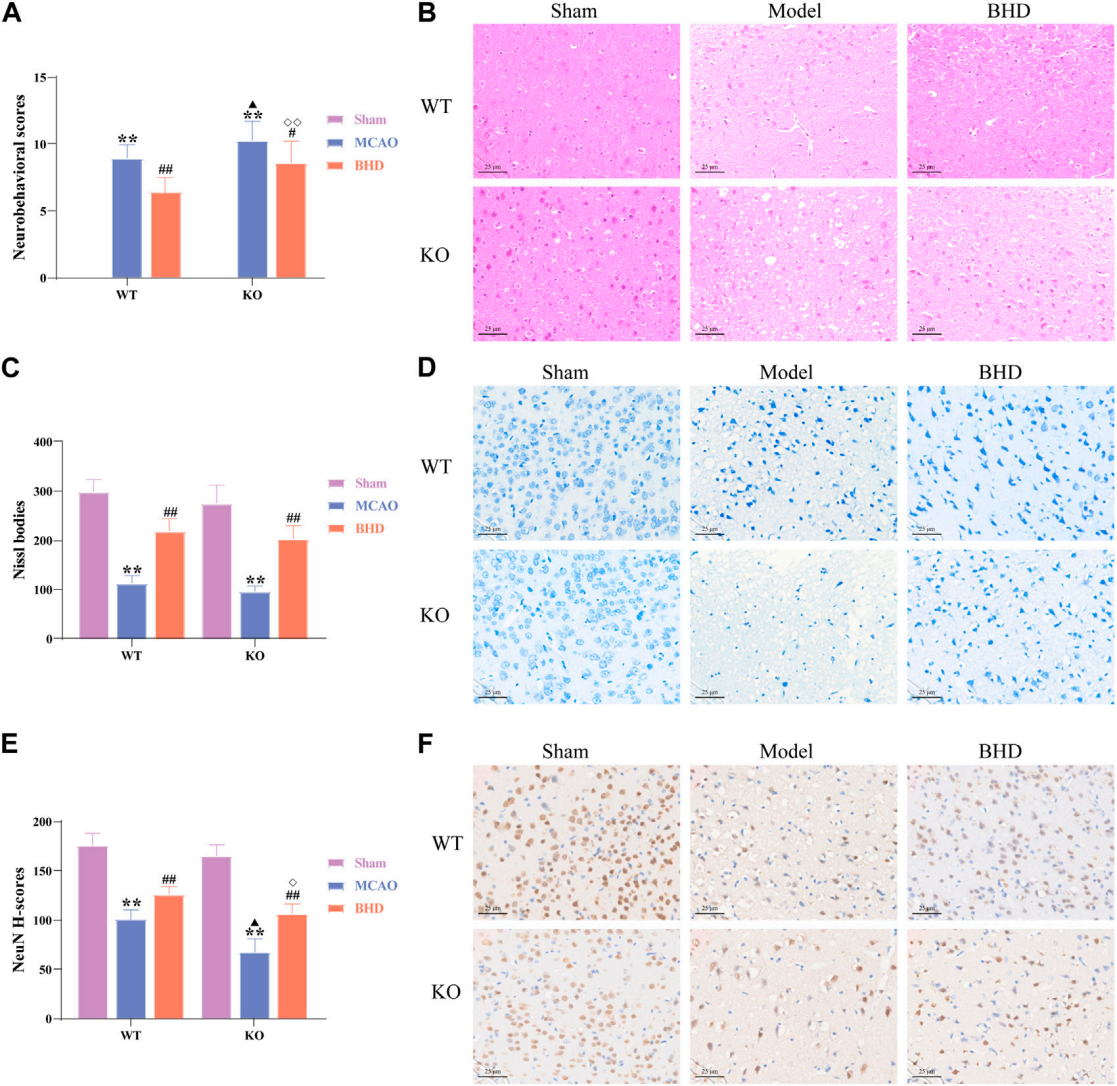
BHD improved neurobehavioral scores and neuronal injury in MCAO mice through Cav-1. **(A)** BHD improved the neurobehavioral score of MCAO mice through Cav-1 (*n* = 12). **(B)** BHD improved the pathological injury of MCAO mice through Cav-1 (*n* = 6). **(C,D)** BHD improved Nissl body injury in MCAO mice through Cav-1 (*n* = 6). **(E,F)** BHD improved neuronal damage in MCAO mice through Cav-1 (*n* = 6). All data are presented as the mean ± SD. **p < 0.01 vs. syngeneic sham group, ^#^ *p* < 0.05 and ^##^ *p* < 0.01 vs. syngeneic model group, ^▲^ *p* < 0.05 vs. WT model group, ^◇^ *p* < 0.05 and ^◇◇^ *p* < 0.01 vs. WT BHD group.

The neurobehavioral score reflected the neurological function of mice in each group. Compared with the syngeneic sham group, the neurobehavioral scores of the WT and KO model groups were significantly increased (*p* < 0.01). Compared with the syngeneic model group, the neurobehavioral scores of the WT and KO BHD groups were significantly decreased (*p* < 0.05 or 0.01). Compared with the WT model group, the neurobehavioral score in the KO model group was significantly higher (*p* < 0.05). Compared with that of the WT BHD group, the neurobehavioral score of the KO BHD group was significantly higher (*p* < 0.01), as shown in Figure 2A. The results showed that Cav-1 deletion increased the neurobehavioral score of MCAO mice, and BHD improved the neurobehavioral score of MCAO mice through Cav-1.

As shown by HE staining (Figure 2B), we found that the cell structure in the sham group was intact, with abundant cytoplasm and clear nuclei. Compared with the syngeneic sham group, the cortical neurons on the ischemic side of the WT and KO model groups were irregularly arranged, with widened intercellular spaces, vacuoles, and pyknosis of nuclei. Compared with the syngeneic model group, the arrangement of cortical neurons on the ischemic side of the BHD group was relatively regular, the intercellular space was reduced, and the nucleolus was clear. Compared with the WT model group, a large number of pyknosis and vacuoles appeared in cortical neurons of the ischemic side in the KO model group. Compared with the WT BHD group, the arrangement of cortical neurons on the ischemic side of the KO BHD group was relatively irregular, and there were more vacuoles.

Nissl bodies serve as a marker of neuronal status. We found that the nerve cells in the sham group were arranged regularly with clear nuclei, abundant Nissl bodies, and uniform staining. Compared with the syngeneic sham group, WT and KO model mice showed significantly sparse cells, hyperchromatic nuclei, and a reduced number of Nissl bodies (*p* < 0.01). Compared with the syngeneic model group, the WT and KO BHD groups showed significantly better cell status, more intact cell morphology and increased Nissl bodies (*p* < 0.01), as shown in Figures 2C, D.

NeuN is often used to label mature neurons. Compared with that in the syngeneic sham group, the expression of NeuN in the WT and KO model groups was significantly decreased (*p* < 0.01). Compared with that in the syngeneic model group, the expression of NeuN in the WT and KO BHD groups was significantly increased (*p* < 0.01). Compared with that in the WT model group, the expression of NeuN in the KO model group was significantly decreased (*p* < 0.05). Compared with that in the WT BHD group, the expression of NeuN in the KO BHD group was significantly decreased (*p* < 0.05), as shown in Figures 2E, F. The above results showed that the deletion of Cav-1 aggravated neuronal damage in MCAO mice and that BHD improved neuronal damage in MCAO mice through Cav-1.

### 3.2 BHD improves mitochondrial morphology in MCAO mice through Cav-1

**FIGURE 3:**
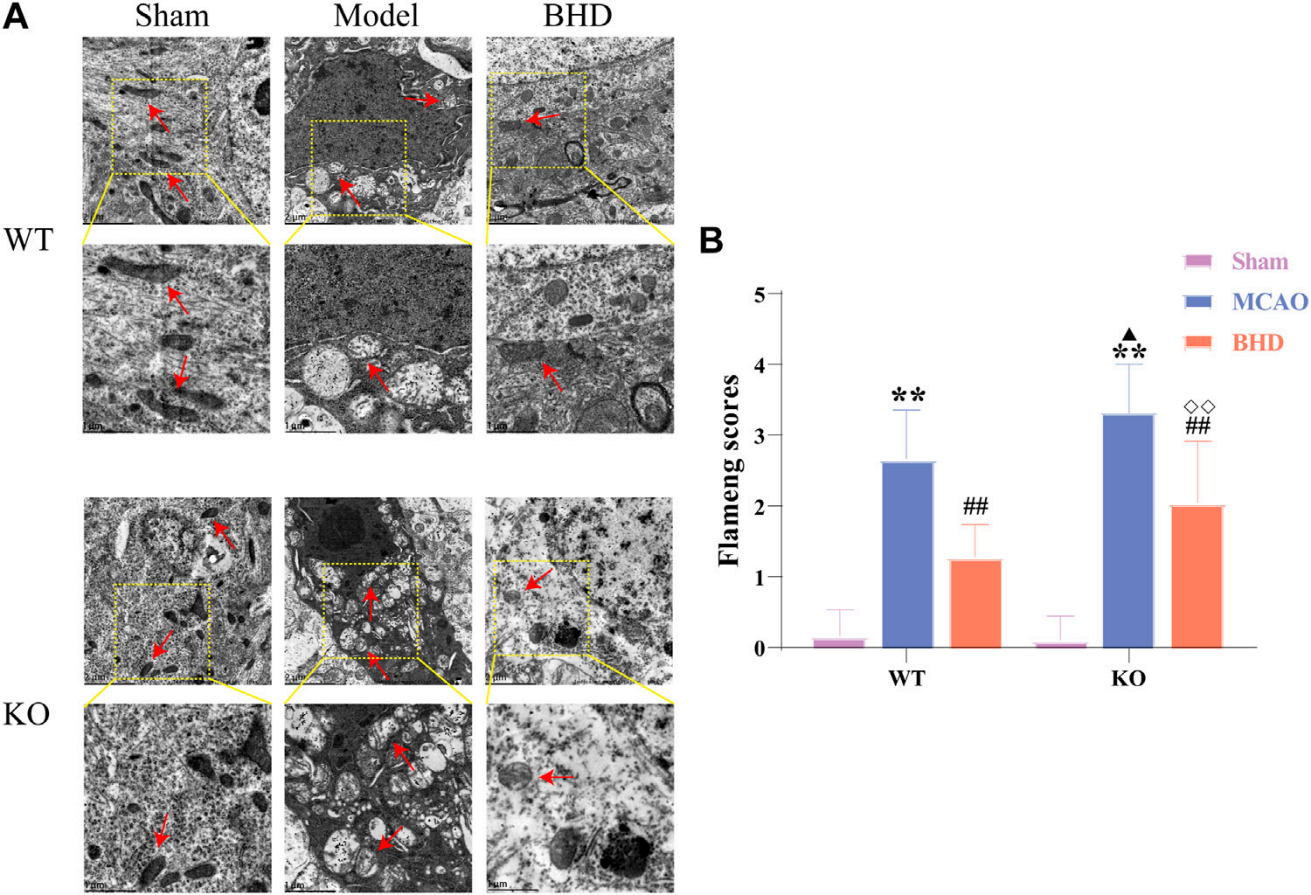
BHD improves mitochondrial morphology in MCAO mice through Cav-1. **(A)** BHD improves mitochondrial morphology in MCAO mice through Cav-1 (*n* = 6). Red arrows indicate mitochondria. **(B)** BHD improves Flameng scores in MCAO mice through Cav-1 (*n* = 6). All data are presented as the mean ± SD. **p < 0.01 vs syngeneic sham group, ^##^ *p* < 0.01 vs syngeneic model group, ^▲^ *p* < 0.05 vs WT model group, ^◇◇^ *p* < 0.01 vs WT BHD group.

The ultrastructure of mitochondria in brain tissue was observed by TEM. In the sham group, the mitochondrial structure was regular and oval, and the inner cristae were clearly visible. Compared with the syngeneic sham group, the mitochondria in the WT and KO model groups were swollen and vacuolated, and the inner cristae structure was fuzzy. Compared with the syngeneic model group, the mitochondria of the WT and KO BHD groups were slightly swollen, and cristae were still present. Compared with the WT model group, the swelling of mitochondria in the KO model group was aggravated, and the cristae were broken or even disappeared. Compared with that of the WT BHD group, the mitochondrial structure of the KO BHD group was blurred, as shown in Figure 3A.

Compared with the syngeneic sham group, the Flameng scores of the WT and KO model groups were significantly increased (*p* < 0.01). Compared with the syngeneic model group, the Flameng scores of the WT and KO BHD groups were significantly decreased (*p* < 0.01). Compared with the WT model group, the Flameng score in the KO model group was significantly higher (*p* < 0.05). Compared with that of the WT BHD group, the Flameng score of the KO BHD group was significantly higher (*p* < 0.01), as shown in Figure 3B. The above results showed that Cav-1 deletion aggravated the damage to mitochondrial morphology in MCAO mice and that BHD improved mitochondrial morphology in MCAO mice through Cav-1.

### 3.3 BHD improves mitochondrial function in MCAO mice through Cav-1

**FIGURE 4:**
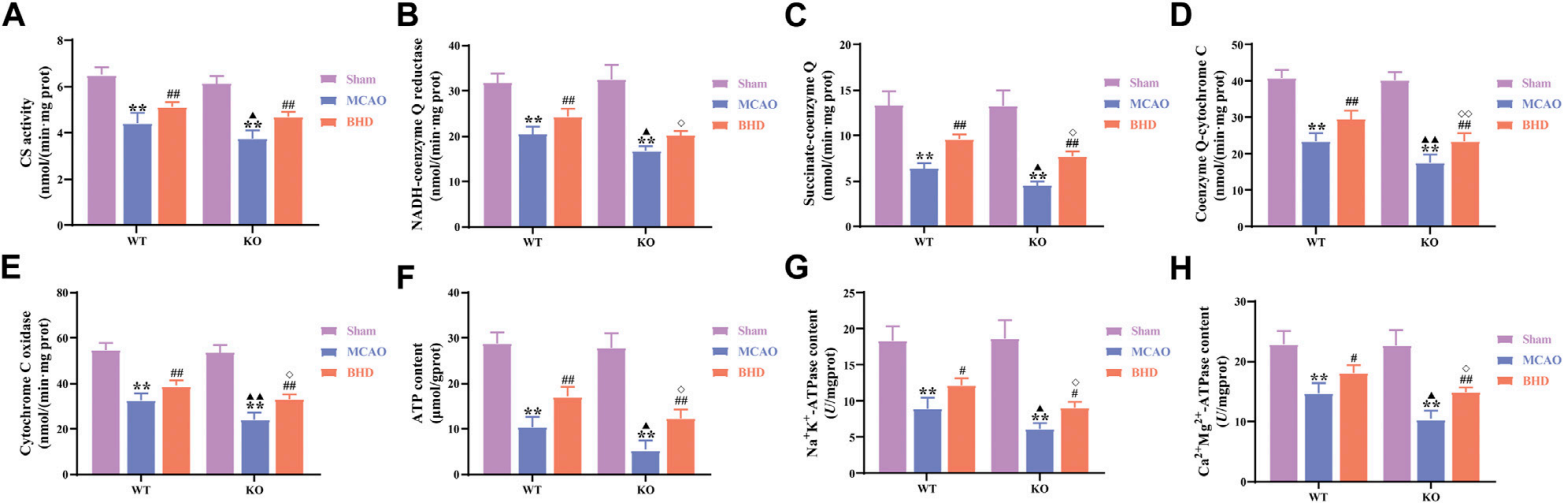
BHD improves mitochondrial function in MCAO mice through Cav-1. **(A)** BHD improves CS activity in MCAO mice through Cav-1 (*n* = 6). **(B)** BHD improves NADH-coenzyme Q in MCAO mice through Cav-1 (*n* = 6). **(C)** BHD improves succinate-coenzyme Q in MCAO mice through Cav-1 (*n* = 6). **(D)** BHD improves coenzyme Q-cytochrome C in MCAO mice through Cav-1 (*n* = 6). **(E)** BHD improves cytochrome C oxidase in MCAO mice through Cav-1 (*n* = 6). **(F)** BHD improves ATP content in MCAO mice through Cav-1 (*n* = 6). **(G,H)** BHD improves ATPase activity in MCAO mice through Cav-1 (*n* = 6). All data are presented as the mean ± SD. **p < 0.01 vs. syngeneic sham group, ^##^ *p* < 0.01 vs. syngeneic model group, ^▲^ *p* < 0.05 and ^▲▲^ *p* < 0.01 vs. WT model group, ^◇^ *p* < 0.05 and ^◇◇^ *p* < 0.01 vs. WT BHD group.

CS activity is closely related to mitochondrial content. Compared with the syngeneic sham group, the CS activity of the WT and KO model groups was significantly decreased (*p* < 0.01). Compared with that of the syngeneic model group, the CS activity of the WT and KO BHD groups was significantly increased (*p* < 0.01). Compared with that in the WT model group, the CS activity in the KO model group was significantly decreased (*p* < 0.05). as shown in Figure 4A.

MRC complexes, ATP and ATPase can reflect mitochondrial function. Compared with the syngeneic sham group, the MRC complex activities, ATP content and ATPase activities of the WT and KO model groups were significantly decreased (*p* < 0.01). Compared with the syngeneic model group, the succinate-coenzyme Q activities, coenzyme Q-cytochrome C activities, cytochrome C oxidase activities, ATP content and ATPase activities of the WT and KO BHD groups were significantly increased (*p* < 0.05 or 0.01). Compared with the WT model group, the MRC complex activities, ATP content and ATPase activities in the KO model group were significantly decreased (*p* < 0.05 or 0.01). Compared with the WT BHD group, the MRC complex activities, ATP content and ATPase activities of the KO BHD group were significantly decreased (*p* < 0.05 or 0.01), as shown in Figures 4B–H. The above results showed that Cav-1 deletion aggravated the damage to mitochondrial function in MCAO mice and that BHD improved mitochondrial function in MCAO mice through Cav-1.

### 3.4 BHD maintains mitochondrial dynamic balance in MCAO mice through Cav-1

**FIGURE 5:**
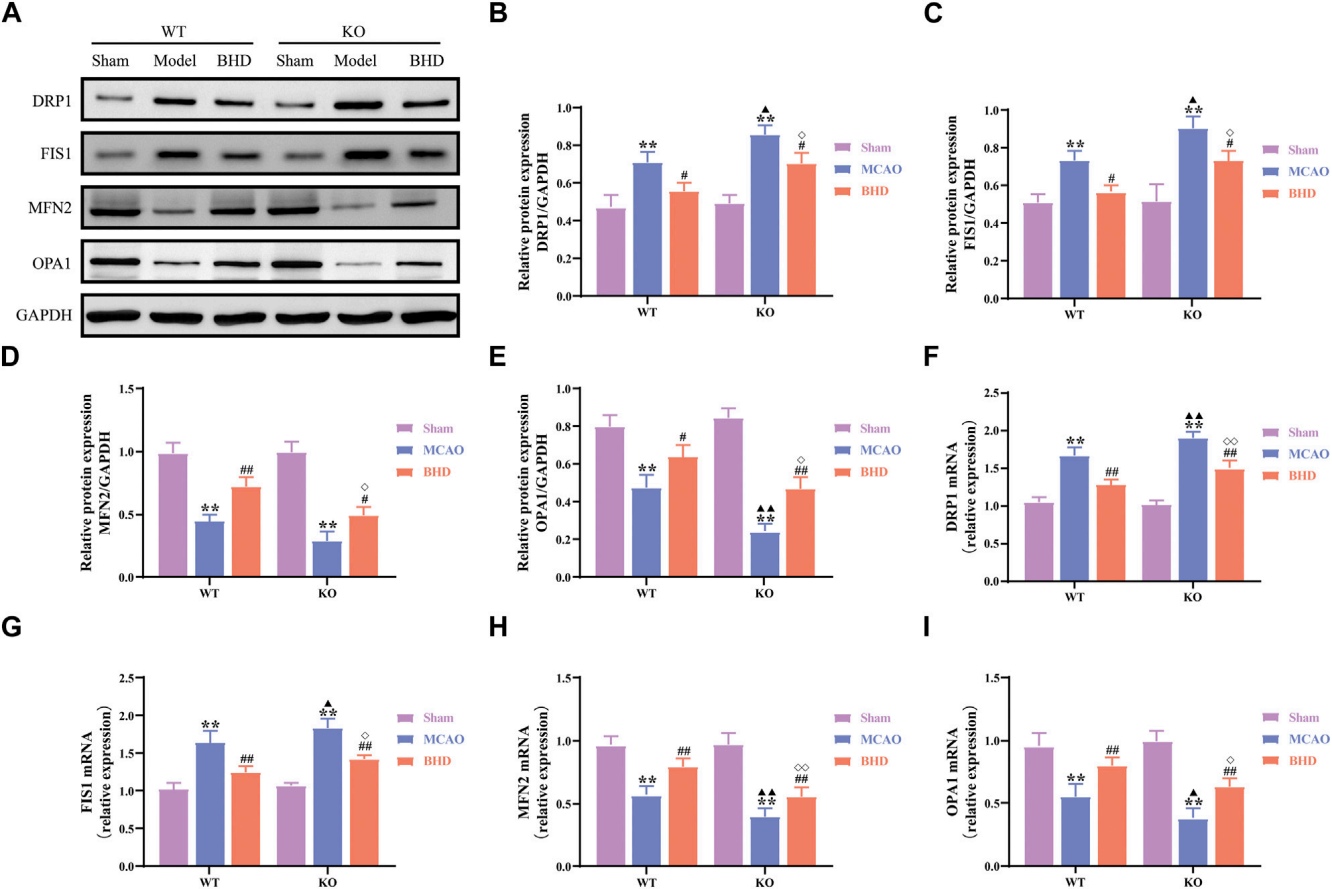
BHD maintains mitochondrial dynamic balance in MCAO mice through Cav-1. **(A–E)** Western blot and quantitative analysis of DRP1, FIS1, MFN2 and OPA1 (*n* = 3). **(F–I)** RT‒qPCR and quantitative analysis of DRP1, FIS1, MFN2 and OPA1 (*n* = 6). All data are presented as the mean ± SD. **p < 0.01 vs. syngeneic sham group, ^#^ *p* < 0.05 and ^##^ *p* < 0.01 vs. syngeneic model group, ^▲^ *p* < 0.05 and ^▲▲^ *p* < 0.01 vs. WT model group, ^◇^ *p* < 0.05 and ^◇◇^ *p* < 0.01 vs. WT BHD group.

The dynamics of mitochondria include mitochondrial fission and fusion. Compared with those in the syngeneic sham group, the protein and mRNA levels of the mitochondrial fission-related factors DRP1 and FIS1 in the WT and KO model groups were significantly upregulated (*p* < 0.01), and the protein and mRNA levels of the mitochondrial fusion-related factors MFN2 and OPA1 were significantly downregulated (*p* < 0.01). Compared with those in the syngeneic model group, the protein and mRNA levels of DRP1 and FIS1 in the WT and KO BHD groups were significantly downregulated (*p* < 0.01 or 0.05), and the protein and mRNA levels of MFN2 and OPA1 were significantly upregulated (*p* < 0.01 or 0.05). Compared with those in the WT model group, the protein and mRNA levels of DRP1 and FIS1 in the KO model group were significantly upregulated (*p* < 0.01 or 0.05), the mRNA levels of MFN2 were significantly downregulated (*p* < 0.01), and the protein and mRNA levels of OPA1 were significantly downregulated (*p* < 0.01 or 0.05). Compared with those in the WT BHD group, the protein and mRNA levels of DRP1 and FIS1 in the KO BHD group were significantly upregulated (*p* < 0.01 or 0.05), and the protein and mRNA levels of MFN2 and OPA1 were significantly downregulated (*p* < 0.01 or 0.05), as shown in Figure 5. The above results showed that the loss of Cav-1 aggravated the imbalance of mitochondrial dynamics in MCAO mice and that BHD could maintain the balance of mitochondrial dynamics in MCAO mice through Cav-1.

### 3.5 BHD promotes mitophagy in MCAO mice through Cav-1

**FIGURE 6:**
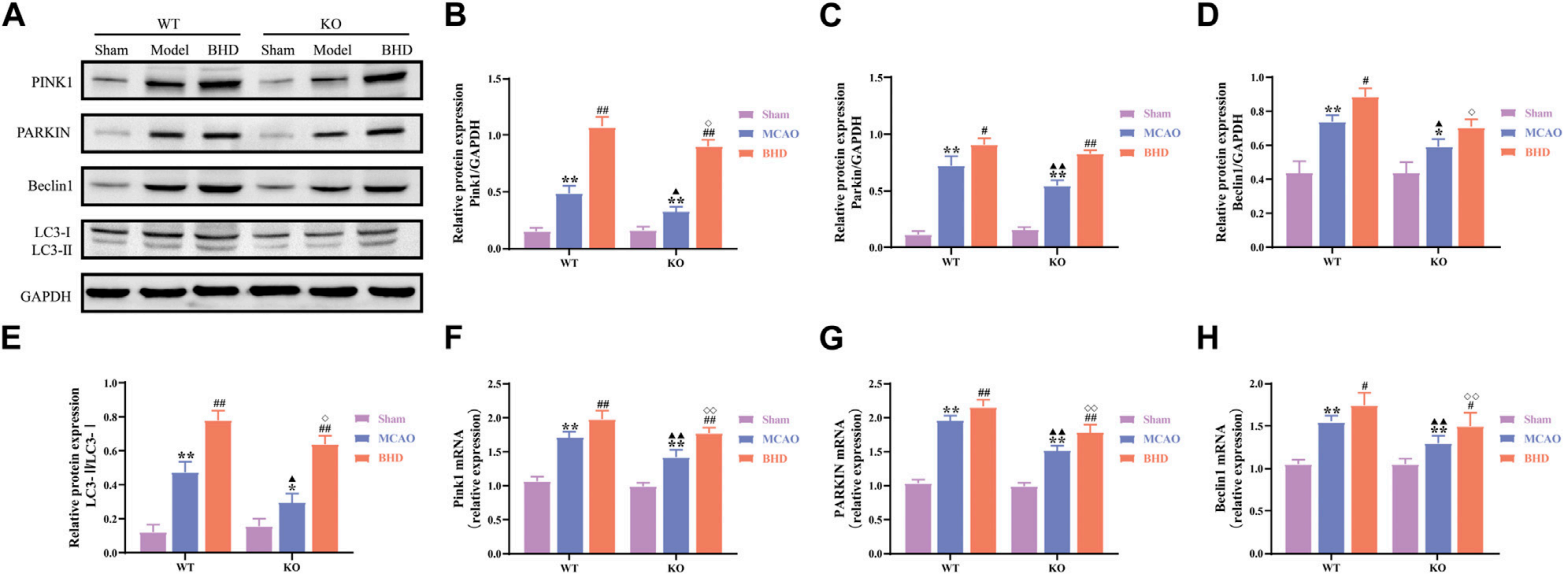
BHD promotes mitophagy in MCAO mice through Cav-1. **(A–E)** Western blot and quantitative analysis of PINK1, PARKIN, Beclin1 and LC3 (*n* = 3). **(F–H)** RT‒qPCR and quantitative analysis of PINK1, PARKIN and Beclin1 (*n* = 6). All data are presented as the mean ± SD. **p < 0.01 vs. syngeneic sham group, ^#^ *p* < 0.05 and ^##^ *p* < 0.01 vs syngeneic model group, ^▲^ *p* < 0.05 and ^▲▲^ *p* < 0.01 vs. WT model group, ^◇^ *p* < 0.05 and ^◇◇^ *p* < 0.01 vs. WT BHD group.

Mitochondria are able to degrade intracellular senescent or damaged mitochondrial fragments or remnants through selective autophagy. Compared with the syngeneic sham group, PINK1, PARKIN, and Beclin1 protein and mRNA levels of mitophagy-related factors in the WT and KO model groups were significantly upregulated (*p* < 0.01 or 0.05), and the LC3-Ⅱ/LC3-Ⅰ ratio was significantly increased (*p* < 0.01 or 0.05). Compared with the syngeneic model group, the protein and mRNA levels of PINK1, PARKIN and the mRNA levels of Beclin1 in the WT and KO BHD groups were further upregulated (*p* < 0.05), the protein levels of Beclin1 in the WT BHD groups were further upregulated (*p* < 0.05), and the ratio of LC3-Ⅱ/LC3-Ⅰ was further increased (*p* < 0.01). Compared with the WT model group, the protein and mRNA expression of PINK1, PARKIN and Beclin1 decreased (*p* < 0.01 or 0.05), and the LC3-Ⅱ/LC3-Ⅰ ratio decreased (*p* < 0.05). Compared with the WT BHD group, the protein and mRNA expression of PINK1 and Beclin1, and the mRNA expression of PARKIN in the KO BHD group decreased (*p* < 0.01 or 0.05), and the LC3-Ⅱ/LC3-Ⅰ ratio decreased (*p* < 0.05), as shown in Figure 6. The above results showed that Cav-1 deletion inhibited mitophagy in MCAO mice and that BHD promoted mitophagy in MCAO mice through Cav-1.

### 3.6 BHD promotes mitochondrial biogenesis in MCAO mice through Cav-1

**FIGURE 7:**
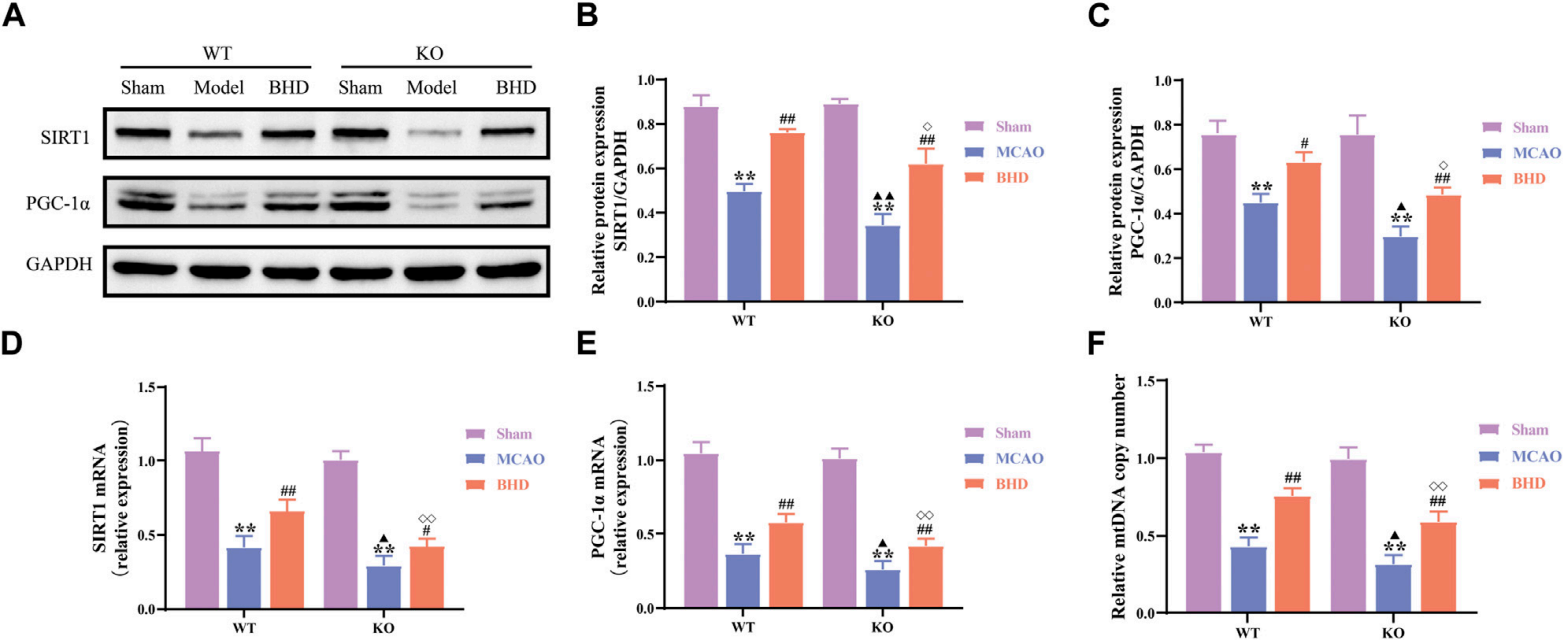
BHD promotes mitochondrial biogenesis in MCAO mice through Cav-1. **(A–C)** Western blot and quantitative analysis of SIRT1 and PGC-1α (*n* = 3). **(D,E)** RT‒qPCR and quantitative analysis of SIRT1 and PGC-1α (*n* = 6) **(F)** BHD promotes mtDNA replication in MCAO mice through Cav-1. All data are presented as the mean ± SD. **p < 0.01 vs. syngeneic sham group, ^#^ *p* < 0.05 and ^##^ *p* < 0.01 vs. syngeneic model group, ^▲^ *p* < 0.05 and ^▲▲^ *p* < 0.01 vs. WT model group, ^◇^ *p* < 0.05 and ^◇◇^ *p* < 0.01 vs. WT BHD group.

Mitochondrial biosynthesis is closely related to mitochondrial functional homeostasis. Compared with those in the syngeneic sham group, the protein and mRNA levels of the mitochondrial biosynthesis-related factors SIRT1 and PGC-1α in the WT and KO model groups were significantly decreased (*p* < 0.01). Compared with the syngeneic model group, the protein levels of SIRT1 and the protein and mRNA levels of SIRT1 and PGC-1α were significantly upregulated in the WT and KO BHD groups (*p* < 0.01 or 0.05). Compared with those in the WT model group, the protein and mRNA levels of SIRT1 and PGC-1α in the KO model group were significantly decreased (*p* < 0.01 or 0.05). Compared with those in the WT BHD group, the protein and mRNA levels of SIRT1 and PGC-1α in the KO BHD group were significantly decreased (*p* < 0.01 or 0.05), as shown in Figures 7A–E.

Mitochondrial biosynthesis also includes tightly regulated mtDNA replication. Compared with the syngeneic sham group, the mtDNA copy number of the WT and KO model groups was significantly decreased (*p* < 0.01). Compared with the syngeneic model group, the mtDNA copy number of the WT and KO BHD groups was significantly increased (*p* < 0.01). Compared with the WT model group, the mtDNA copy number in the KO model group was significantly decreased (*p* < 0.05). Compared with the WT BHD group, the mtDNA copy number of the KO BHD group was significantly decreased (*p* < 0.01), as shown in Figure 7F. These results suggest that Cav-1 deficiency inhibits mitochondrial biosynthesis in MCAO mice and that BHD can promote mitochondrial biosynthesis in MCAO mice through Cav-1.

## 4 Discussion

After CI, mitochondria produce a large number of reactive oxygen species and undergo an oxidative stress response, resulting in calcium overload and continuous accumulation of proinflammatory factors damaging mitochondria, causing mitochondrial swelling and energy synthesis disorders and triggering programmed cell death, resulting in irreversible damage (Song et al., 2022). However, MQC can clear the internal structure of irreversible damage through autophagy, maintain the morphology of mitochondria and increase energy supply to stabilize themselves and protect nerve cells through biosynthesis and fusion, which is currently a research hotspot (Yang et al., 2021).

Neurons are the most important cells in the central nervous system (CNS), and they are also the key for the CNS to perform normal neurotransmission and signaling (Fricker et al., 2018). Studies have shown that Cav-1 is expressed in normal neurons and senescent neurons and plays a positive regulatory role in neuronal plasticity and neural stem cell proliferation and differentiation after nervous system injury (Gaudreault et al., 2005). Cav-1 overexpressing mice showed better neurological function recovery after traumatic brain stimulation than normal mice, and the brain injury volume was smaller (Mandyam et al., 2017; Egawa et al., 2018). Cav-1 protects neurons from ischemic injury and inhibits cell death by regulating the extracellular regulated kinase (ERK) 1/2 signaling pathway (Zhong et al., 2020). In the present study, we found that mice developed neurological and neuronal impairment after CI and caused an imbalance in the MQC and that knockout of Cav-1 aggravated neurological function and neuronal damage and disturbed MQC after CI. BHD may play an anti-CI role by regulating MQC through Cav-1. To our knowledge, this is the first report on the effect of BHD and Cav-1 on MQC after CI.

In this study, we found that the structure of mitochondria was significantly damaged and the activities of CS, MRC complexes, ATP and ATPase were significantly decreased after CI, which was consistent with a previous report (Gibbs et al., 2016). The intervention of BHD reversed the above changes. Notably, the depletion of Cav-1 aggravated mitochondrial functional damage and inhibited the therapeutic effect of BHD. It has been confirmed that Cav-1 deficiency leads to the accumulation of lipids in the mitochondria, which results in reduced mitochondrial adaptation to hypoxia and aggravates inflammation and oxidative stress (Bosch et al., 2011; Powter et al., 2015), but the potential mechanism remains unclear. In summary, the current data reveal that Cav-1 may affect the structure and function of mitochondria after CI and that BHD may maintain the structure and function of mitochondria in MCAO mice through Cav-1. In view of the potential key roles of BHD and Cav-1 in the regulation of mitochondrial function after CI, we further analyzed the effects of BHD and Cav-1 on MQC after CI.

MQC includes mitochondrial fusion, division, autophagy and biosynthesis, and its various links coordinate to maintain the normal function of mitochondria. Mitochondria guarantee that cellular energy demand mainly depends on their fusion and division functions, and the antagonistic balance between them is the key to maintaining MQC and ensuring cellular energy metabolism (Song et al., 2015). Mitochondrial fusion is divided into mitochondrial outer membrane fusion and inner membrane fusion. In mammals, MFN1 and MFN2 promote mitochondrial outer membrane fusion through mitochondrial cristae morphological remodeling, and OPA1 mediates mitochondrial inner membrane fusion to form a reticular structure to improve the speed of energy synthesis (Cretin et al., 2021). Mitochondrial fission is mainly regulated by DRP1 and FIS1. DRP1 can be recruited to the outer mitochondrial membrane through FIS1 and a variety of other adaptor proteins, forming a ring around the mitochondria and then winding and contracting to turn the protomitochondria into two separate mitochondria (Schmitt et al., 2018). Relevant studies have confirmed that after CI, mitochondrial fission is significantly increased, while mitochondrial fusion is significantly reduced. By inhibiting mitochondrial fission and promoting mitochondrial fusion, CI injury can be slowed (Meyer et al., 2017). This study confirmed that the protein and mRNA expression levels of FIS1 and DRP1 were significantly increased in MCAO mice, while the levels of MFN2 and OPA1 were decreased, indicating that the mitochondrial dynamics (division/fusion) of the ischemic side of brain tissue in mice with CI appeared to be obviously imbalanced. BHD not only reduced the expression of FIS1 and DRP1 but also increased the expression levels of MFN2 and OPA1, suggesting that BHD can repair the imbalance in mitochondrial dynamics after CI. In addition, we also found that Cav-1 knockdown upregulated the expression of FIS1 and DRP1 and inhibited the expression levels of MFN2 and OPA1. At present, some researchers have found that Cav-1 can regulate the recruitment of mitochondrial dynamics-related proteins in tumor cells and regulate mitochondrial dynamics balance (Xiao et al., 2022). The above results indicated that Cav-1 may regulate mitochondrial dynamic balance after CI and that BHD may maintain mitochondrial dynamic balance in MCAO mice through Cav-1.

Mitophagy, which maintains cellular homeostasis by selectively scavenging damaged or dysfunctional mitochondria, is a key link in regulating MQC (Ktistakis, 2021). Due to the special morphology of neurons, mitochondria are mainly distributed in elongated axons and soma, while lysosomes are concentrated in soma. Studies have found that the mitochondria of axons after CI are transported into the soma in reverse, which in turn initiates mitophagy and alleviates mitochondrial dysfunction and neuronal damage (Zheng et al., 2019). The PINK1/PARKIN pathway is the classical pathway of mitophagy (Bakula and Scheibye-Knudsen, 2020). When mitochondria are damaged and the protease is inactivated, PINK1 not only phosphorylates itself to recognize ubiquitin but also recruits and activates E3-ubiquitin ligase, which rapidly encircles damaged mitochondria and enhances ubiquitin chain activity on the membrane. It promotes the binding of ubiquitinated proteins to adaptor proteins and LC3 to form autophagosomes and initiate autophagy (Zhang et al., 2021). Some studies have found that the PINK1/PARKIN-mediated mitophagy pathway is activated after CI, and mitochondrial division inhibitors aggravate ischemia-induced nerve cell damage, suggesting that promoting mitophagy has a protective role in CI injury (He et al., 2019). In this study, we found that PINK1, PARKIN and Beclin1 were significantly upregulated and that the LC3-Ⅱ/LC3-Ⅰ ratio was significantly increased in MCAO mice, suggesting that mitophagy occurs after CI. However, the level of spontaneous mitophagy is weak enough to completely remove damaged mitochondria, and BHD can significantly improve the level of mitophagy and reduce CI injury. Interestingly, we also found that Cav-1 knockout inhibited mitophagy in MCAO mice, which is consistent with previous reports that Cav-1 promotes mitophagy (Jiang et al., 2021). The above results showed that Cav-1 could regulate mitophagy after CI and that BHD might play an anti-CI role by promoting mitophagy in MCAO mice through Cav-1.

Mitochondrial biogenesis is regulated by mtDNA and its nuclear DNA to complete mitochondrial proliferation and then produce new mitochondria to meet the energy needs of cells. The self-renewal of mitochondria through mitochondrial biogenesis is very important for maintaining the integrity of mitochondria and cellular energy metabolism (Liang et al., 2021). PGC-1α is a core molecule of mitochondrial biogenesis (Koh and Kim, 2021). As a major regulator of mitochondrial biogenesis, PGC-1α can be deacetylated by the upstream SIRT1 protein to promote mtDNA replication, transcription and translation (Scarpulla, 2008). In this study, SIRT1, PGC-1α and mtDNA copy number were significantly decreased after CI, and BHD intervention reversed the above changes. In addition, Cav-1 knockdown suppressed SIRT1 and PGC-1α expression and reduced mtDNA copy number. These results suggest that Cav-1 deficiency inhibits mitochondrial biosynthesis in MCAO mice and that BHD can promote mitochondrial biosynthesis in MCAO mice through Cav-1.

**FIGURE 8:**
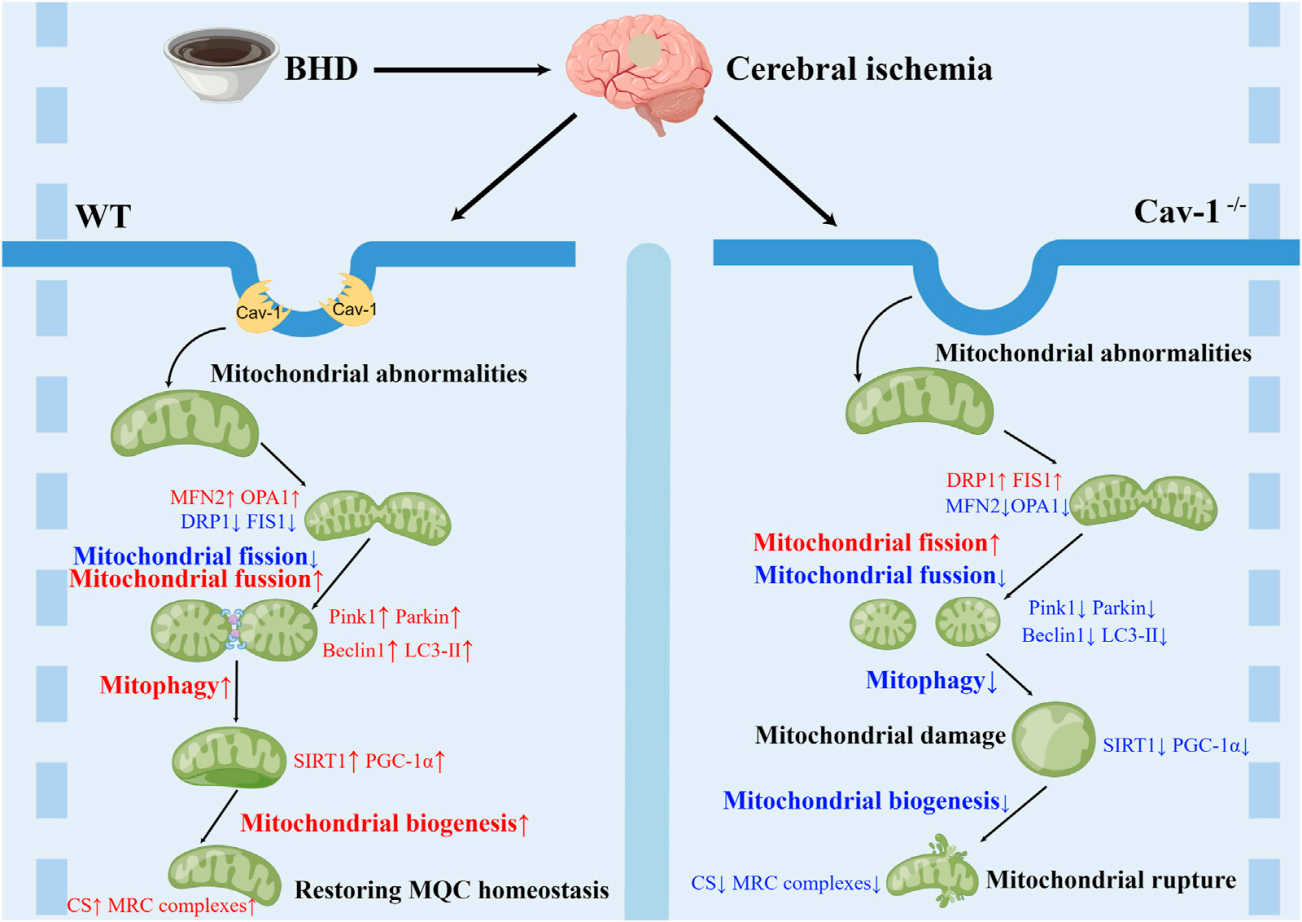
Schematic diagram of the mechanism by which BHD improves CI.

Collectively, we demonstrate a clear correlation between Cav-1 and mitochondria in the nervous system after CI. Cav-1 deficiency aggravates the MQC imbalance after CI, aggravates the functional damage to mitochondria and inhibits the therapeutic effects of BHD. Based on the current reports, the mechanism may be associated with Cav-1 promoting the recruitment of mitochondrial dynamics-related proteins to mitochondria (Xiao et al., 2022) and regulating lipid transport in mitochondria (Bosch et al., 2011), which needs to be further investigated. However, it is worth noting that some studies suggest that BHD treatment can reduce Cav-1 expression (Chen et al., 2020), which may be caused by different species, detection times and detection sites. In addition, the lack of *in vitro* mitochondrial experiments to evaluate the drug effect in this study is not helpful to explain the direct mechanism of drug action. In addition, mitochondrial dynamics and autophagy are continuous dynamic processes, and static evaluation of the role of independent links of MQC and their mechanisms at a single time point may generate research bias. It may be necessary to clarify the spatiotemporal evolution of Cav-1 after CI through single-cell sequencing or spatial transcriptome technology on the basis of multiple time points, which will be the focus of our future studies. However, in general, the present study found that BHD exerted its anti-ischemic effect probably through Cav-1-mediated regulation of MQC (Figure 8).

## 5 Conclusion

In summary, the present study found that the imbalance of MQC after CI leads to the damage to mitochondrial structure and function, and Cav-1 can regulate MQC after CI, suggesting that Cav-1 may be a potential target for the treatment of CI. In addition, we also suggested that BHD exerted its anti-ischemic effect possibly by regulating MQC through Cav-1.

## Data Availability

The original contributions presented in the study are included in the article/Supplementary Materials, further inquiries can be directed to the corresponding authors.
