# Supplementary material for: Buyang Huanwu Decoction alleviates cerebral ischemic injury through modulating caveolin-1-mediated mitochondrial quality control
Source: Front Pharmacol. 2023 May 10;14:1137609. doi: 10.3389/fphar.2023.1137609 (PMC10206009; doi:10.3389/fphar.2023.1137609)
Supplement: Supplementary file 2 [file DataSheet1.PDF]

Genotyping Report

Strain ID	T010293	Strain Type	KO(Cas9)	Genetic Background	C57BL/6JGpt
Designer	Zifan Lin	Gene Name	*Cav1*		

1. Strategy of Genotyping

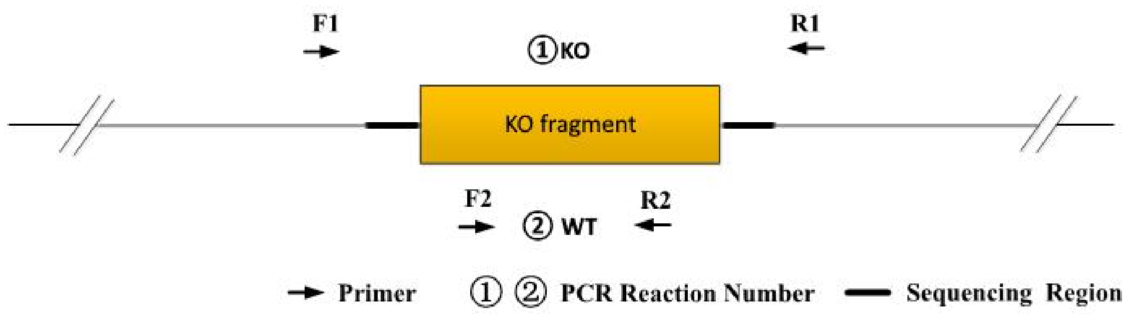

Wild type: ①PCR reaction obtains a single WT band; ②PCR reaction obtains a single WT band.
Heterozygote: ①PCR reaction obtains a WT band and a KO band; ②PCR reaction obtains a WT band.
Homozygote: ①PCR reaction obtains a single KO band; ② PCR reaction without product.
Note：1)The sizes of WT and Targeted band are shown below.
2)If the WT band is too large, it may not be possible to obtain a WT band.

2. Primer Information

PCR No.	Primer No.	Sequence	Band Size
PCR①	T010293-F1	GAGTTAATAGAAGGGCAAAGGCTGT	WT:8157bp<br>Targeted:244bp
	T010293-R1	CATCACCTCCTCAATGGTGAATCA	
PCR②	T010293-F2	GGGCATGACATGCCTATAAATTCC	WT:293bp<br>Targeted:0bp
	T010293-R2	CTGACTGAAGATTTGTTACCTTGCTG	

3. Gel Image

acatttgcaatttgagttagctggtttgttccgac-------*7913bp*------actcgagctgtccttggcagaggatttcctctgag

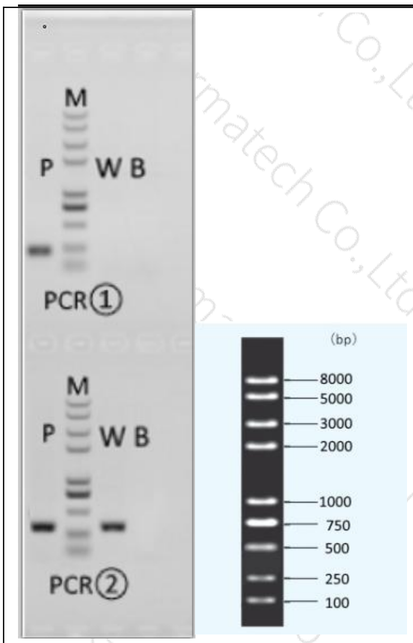

Note: P: Positive control; WT: Wildype control; B: Blank control (ddH2O); M: DNA Ladder

4. PCR Condition

PCR Reaction Component		
**Seg.**	**reaction component**	**Volume (μl)**
1	2 × Taq Master Mix， Dye Plus，(Vazyme P112-03)	12.5
2	ddH2O	9.5
3	Primer A(10pmol/μl)	1
4	Primer B(10pmol/μl)	1
5	Template(≈100ng/μl)	1

PCR program			
**Seg.**	**Temp.**	**Time**	**Cycle**
1	95℃	5min	
2	98℃	30s	20×
3	65℃（-0.5℃/cycle）	30s	
4	72℃	45s	
5	98℃	30s	20×
6	55℃	30s	
7	72℃	45s	
8	72℃	5min	
9	10℃	hold	

Reviewer:Ting Sun

Date:2021-5-24

GemPharmatech Co.,Ltd
